# Cardiac-sparing radiotherapy for locally advanced non-small cell lung cancer

**DOI:** 10.1186/s13014-021-01824-3

**Published:** 2021-06-03

**Authors:** Louise Turtle, Neeraj Bhalla, Andrew Willett, Robert Biggar, Jonathan Leadbetter, Georgios Georgiou, James M. Wilson, Sindu Vivekanandan, Maria A. Hawkins, Michael Brada, John D. Fenwick

**Affiliations:** 1 Department of Radiotherapy, The Clatterbridge Cancer Centre NHS Foundation Trust, Bebington, CH63 4JY Wirral UK; 2 Medical Physics, Royal Devon and Exeter NHS Foundation Trust, Exeter, EX2 5DW UK; 3 Molecular and Clinical Cancer Medicine, Institute of Systems, Molecular and Integrative Biology, University of Liverpool, Royal Liverpool University Hospital, Liverpool, L69 3GA UK; 4 Medical Physics and Biomedical Engineering, University College London, Gower Street, London, WC1E 6BT UK; 5 University College London Hospital NHS Foundation Trust, 235 Euston Road, London, NW1 2BU UK; 6 Guy’s and St. Thomas’ NHS Foundation Trust, Westminster Bridge Road, London, SE1 7EH UK

**Keywords:** NSCLC, Cardiac-sparing, Radiotherapy, Heart, Survival

## Abstract

**Background:**

We have carried out a study to determine the scope for reducing heart doses in photon beam radiotherapy of locally advanced non-small cell lung cancer (LA-NSCLC).

**Materials and methods:**

Baseline VMAT plans were created for 20 LA-NSCLC patients following the IDEAL-CRT isotoxic protocol, and were re-optimized after adding an objective limiting heart mean dose (MD_Heart_). Reductions in MD_Heart_ achievable without breaching limits on target coverage or normal tissue irradiation were determined. The process was repeated for objectives limiting the heart volume receiving ≥ 50 Gy (V_Heart-50-Gy_) and left atrial wall volume receiving ≥ 63 Gy (V_LAwall-63-Gy_).

**Results:**

Following re-optimization, mean MD_Heart_, V_Heart-50-Gy_ and V_LAwall-63-Gy_ values fell by 4.8 Gy and 2.2% and 2.4% absolute respectively. On the basis of associations observed between survival and cardiac irradiation in an independent dataset, the purposefully-achieved reduction in MD_Heart_ is expected to lead to the largest improvement in overall survival. It also led to useful knock-on reductions in many measures of cardiac irradiation including V_Heart-50-Gy_ and V_LAwall-63-Gy_, providing some insurance against survival being more strongly related to these measures than to MD_Heart_. The predicted hazard ratio (HR) for death corresponding to the purposefully-achieved mean reduction in MD_Heart_ was 0.806, according to which a randomized trial would require 1140 patients to test improved survival with 0.05 significance and 80% power. In patients whose baseline MD_Heart_ values exceeded the median value in a published series, the average MD_Heart_ reduction was particularly large, 8.8 Gy. The corresponding predicted HR is potentially testable in trials recruiting 359 patients enriched for greater MD_Heart_ values.

**Conclusions:**

Cardiac irradiation in RT of LA-NSCLC can be reduced substantially. Of the measures studied, reduction of MD_Heart_ led to the greatest predicted increase in survival, and to useful knock-on reductions in other cardiac irradiation measures reported to be associated with survival. Potential improvements in survival can be trialled more efficiently in a population enriched for patients with greater baseline MD_Heart_ levels, for whom larger reductions in heart doses can be achieved.

**Supplementary Information:**

The online version contains supplementary material available at 10.1186/s13014-021-01824-3.

## Background

Radical chemoradiotherapy (CRT) is the standard-of-care for patients with inoperable locally-advanced non-small cell lung cancer (LA-NSCLC). In a meta-analysis, improved overall survival (OS) following radiotherapy (RT) alone or sequential CRT was associated with increased tumour radiation doses [1]. For concurrent CRT, however, survival was significantly shorter in the high-dose arm of the Radiation Therapy Oncology Group (RTOG)-0617 randomized trial of 74 Gy versus 60 Gy [2].

The RTOG-0617 finding might be explained by survival-limiting toxicities at higher dose-levels. Analysis of data from the IDEAL-CRT trial demonstrated a significant negative association between OS and left atrial (LA) wall volumes receiving radiation doses ≥ 63 Gy in LA-NSCLC patients treated using concurrent CRT [3]. Similarly, in patients treated routinely with RT ± induction chemotherapy, OS was negatively associated with doses delivered to the base of heart, a region formed by the two atria [4]. And in RTOG-0617 patients, OS was also negatively associated with cardiac irradiation [2].

Difficulties distinguishing deaths related to radiation-induced heart disease (RIHD) from cancer-related deaths make it challenging to determine whether these associations are causal. Furthermore, causal explanations other than RIHD are possible, for example an elevated neutrophil-to-lymphocyte ratio resulting from heart irradiation [5]. Non-causal explanations have also been proposed, such as associations between heart doses and the location of involved mediastinal nodes [6], previously found to affect survival. However, in a multivariable analysis of survival in IDEAL-CRT, heart irradiation remained independently significantly associated with OS even when N2/3 disease and subcarinal nodal involvement were included in the analysis [3]. A randomized trial of cardiac-sparing RT would potentially provide the clearest demonstration of a causal link between heart doses and survival for LA-NSCLC patients.

Here, we determine the extent to which heart doses can be reduced. Since limits placed on heart doses have been met easily in many trials [2, 7] we have investigated lower and more challenging limits within an existing dose-escalation study design. Using the CT scans of patients who had received routine treatment, new baseline plans were created representing the treatments these patients would have received in the IDEAL-CRT study, in which tumour doses of 63–73 Gy in 30 fractions were prescribed isotoxically [8]. Then we determined by how much heart irradiation could be reduced without breaching protocol limits on irradiation of other organs-at-risk (OARs) or dose-coverage of planning and clinical target volumes (PTV/CTVs).

Because the cardiac irradiation measure most predictive of shorter survival has yet to be conclusively identified, we tested the feasibility of reducing three measures reported to be associated with OS or risk of major coronary events: heart mean dose (MD_Heart_) [9, 10]; the whole-heart fractional volume receiving ≥ 50 Gy (V_Heart-50-Gy_) [11]; and the LA wall volume receiving ≥ 63 Gy (V_LAwall-63-Gy_) [3]. We have also investigated the degree to which reductions made purposefully in these three measures generate knock-on reductions in the others, and the additional knock-on reductions they generate in doses delivered to the right atrium, left and right ventricles, aortic valve, ascending aorta and right coronary artery, which have also been found to be associated with survival [12–14]. Finally, expected improvements in OS were calculated for the mean reductions achieved in MD_Heart_, V_Heart-50-Gy_ and V_LAwall-63-Gy_, and used to estimate numbers of patients that would be needed to detect survival improvements in randomized trials.

## Methods

Study plans were created with institutional approval for 20 anonymized LA-NSCLC patients, 12 stage IIIA and 8 IIIB with an equal split of left- and right-sided disease, treated at Clatterbridge Cancer Centre (CCC) during 2016–2017 (Additional file 1: Table S1). Internal gross tumour volumes (iGTVs) were defined by drawing contours on 4D-CT average-intensity projections (AIPs), and were expanded by 5 mm to form clinical target volumes (CTVs) and another 5 mm to form PTVs. OAR contours were also drawn on the AIPs. Heart outlines were drawn according to SCOPE-1 and IDEAL-CRT study guidelines [15]. Delineation of cardiac structures was guided by published atlases [16, 17], with LA wall defined as the region ≤ 5 mm within the LA contour [3] and the aortic valve region as the valve plus 5 mm to allow for movement [12].

Treatments were planned in Eclipse version 13.6 (Varian Medical Systems, Palo Alto, Ca) using the Acuros dose algorithm. Baseline dual-arc VMAT plans covered 99% of the CTV and 90% of the PTV with ≥ 95% of the prescribed dose, and 98% of the PTV with ≥ 90% of this dose [8]. Doses were prescribed to the median PTV level, and initially selected so that the mean equivalent dose in 2 Gy fractions across both lungs minus iGTV (EQD2_Lung-mean_, α/β = 3 Gy) was 16.5 Gy for each patient. Then they were limited to 63–73 Gy and reduced if necessary to meet the IDEAL-CRT normal tissue dose-volume limits listed in Additional file 1: Table S2 [8]. Reflecting routine CCC practice, optimization included an objective with a priority level of 100 to minimize cardiac hot-spots above the prescribed dose-level, and further objectives whose priorities were raised from 50 if cardiac dose-volume measures exceeded protocol limits.

These plans were re-optimized, raising the prioritization of an additional penalty designed to reduce MD_Heart_, and determining the maximum reduction in this index achievable without changing the prescribed dose or violating the coverage constraints or OAR dose-volume limits of Additional file 1: Table S2. This process was repeated, re-optimizing using new penalties designed to lessen V_Heart-50-Gy_ and V_LAwall-63-Gy_.

For the baseline and re-optimized plans, target volume coverage-levels were noted together with values of MD_Heart_, V_Heart-50-Gy_, V_LAwall-63-Gy_ and the OAR dose-volume measures of Additional file 1: Table S2. Mean physical doses in the LA wall, aortic valve region and lungs minus iGTV were also noted, together with mean and maximum doses in the right atrium and both ventricles, ascending aorta and right coronary artery. Volumes of lungs minus iGTV receiving ≥ 10, 30 and 50 Gy (V_Lung-10,30,50-Gy_) and the aortic valve region receiving 35–43 Gy (V_AVR-35–43-Gy_) were also recorded.

To contextualize cardiac irradiation-levels, we identified targets for the purposefully reduced dose-volume measures. For MD_Heart_, basic, moderate and ambitious target-levels were defined as 20, 11 and 5 Gy, corresponding to roughly the 85^th^, 50^th^ and 20^th^ MD_Heart_ percentiles in a patient cohort in which 2-year cumulative incidence of grade ≥ 3 cardiac events was 2% in patients with MD_Heart_ ≤ 11 Gy versus 18% in others [10]. For V_Heart-50-Gy_, analogous levels of 25%, 4% and 0.5% were identified. The first reflects results from a study in which 2-year OS was 20% higher for patients with V_Heart-50-Gy_ < 25% than for others [11]. The latter two were the 50^th^ and 20^th^ percentiles of V_Heart-50-Gy_ values in IDEAL-CRT. For V_LAwall-63-Gy_, levels of 20%, 2.2% and 0% were identified, roughly the 85^th^, 50^th^ and 33^rd^ V_LAwall-63-Gy_ percentiles in IDEAL-CRT patients, amongst whom 2-year survival was 23% higher in patients with V_LAwall-63-Gy_ < 2.2% than in others [3].

Reduced heart irradiation might be accompanied by diminished target volume coverage or increased irradiation of other OARs, even while remaining within protocol limits. Details of any lessening of coverage are provided, together with changes in lung irradiation. Significances of changes in distributions of these measures were assessed using the two-sided Wilcoxon signed-rank test. Changes in numbers of patients with OARs lying within 10% of protocol dose-volume limits are also tabulated.

In an independent patient cohort, analysed to validate associations between heart dosimetry and OS seen in the IDEAL-CRT study, hazard ratios (HRs) for all-cause death were 0.956 per 1 Gy decrease in MD_Heart_, 0.974 per 1% absolute decrease in V_Heart-50-Gy_, and 0.929 per 1% decrease in a measure equivalent to V_LAwall-63-Gy_ allowing for a small change in fractionation [3, 12]. We translated these values into HRs for the mean reductions in cardiac irradiation achieved in this study. On the basis of the resulting HRs and survival in IDEAL-CRT [18], we have estimated numbers of patients that would be needed for trials designed to test improved OS with a 5% type-I error rate and 80% power, if randomized 1:1 and with 3 years’ recruitment and 2 years’ further followup [19].

## Results

### Patients and baseline plans

**Fig. 1:**
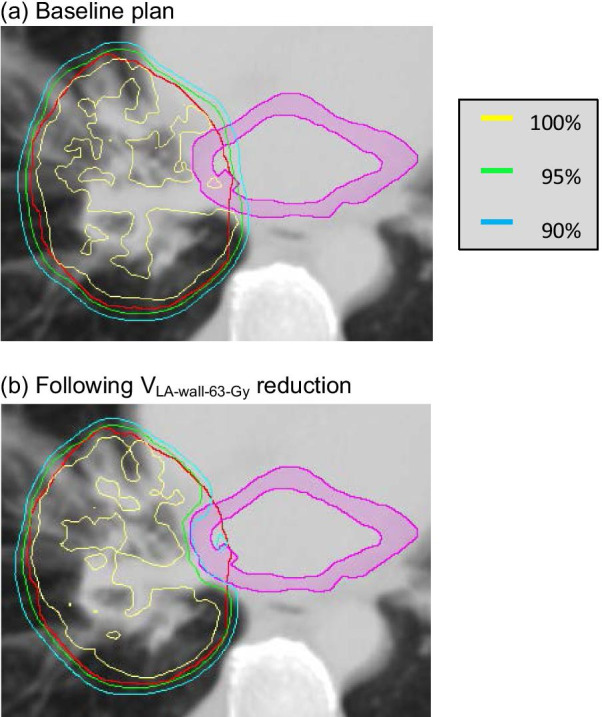
PTV contour and LA wall in a patient with a 3.9 cm^3^ PTV/LA wall overlap. The PTV is shown in red, and the LA wall structure in pink. Isodose lines representing 68.8, 65.2 and 61.9 Gy (100%, 95% and 90% of the prescribed dose) are plotted **a** at baseline and **b** after re-optimization to reduce V_LAwall-63-Gy_

For the patients studied, disease stage, prescribed dose and tumour geometric characteristics are listed in Additional file 1: Table S1. For the iGTV, the median volume (range) was 106.0 cm^3^ (7.4, 243.2 cm^3^). For the CTV and PTV the median volumes (ranges) of overlaps with the heart were 1.3 cm^3^ (0, 19.9 cm^3^) and 8.3 cm^3^ (0, 42.3 cm^3^) respectively, two patients having no CTV overlap with the heart and one no PTV overlap. The median volume (range) of overlaps between the PTV and LA wall was 0.1 cm^3^ (0, 4.1 cm^3^) with no overlap in 10 patients. Figure 1 shows a CT slice from a patient with a 3.9 cm^3^ PTV/LA wall overlap.

The median (range) of prescribed doses was 68.8 Gy (63.0, 73.0 Gy). IDEAL-CRT target volume coverage requirements and OAR irradiation limits were met in baseline plans. The limits on heart irradiation were met particularly easily: median values of the minimum doses to the most highly irradiated 100%, 67% and 33% of the heart were 0.6, 1.9 and 4.5 Gy compared to limits of 45, 53 and 60 Gy (Additional file 1: Table S3).

### ***Purposeful MD***_***Heart***_*** reductions***

**Fig. 2:**
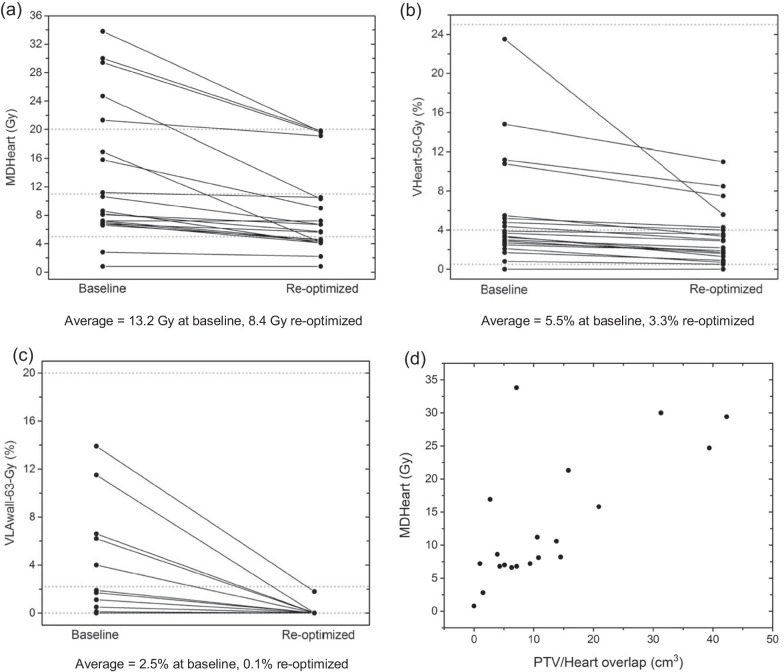
Cardiac dose-volume measures plotted for baseline and re-optimized plans. The re-optimized plans were designed to reduce **a** MD_Heart_, **b** V_Heart-50-Gy_ and **c** V_LAwall-63-Gy_. Basic, moderate and ambitious target levels for the different dose-volume measures are shown as dotted lines. In **d** baseline MD_Heart_ values are plotted against the PTV/Heart overlap

MD_Heart_ values in baseline plans and plans re-optimized to reduce this measure are plotted in Fig. 2a. The average MD_Heart_ reduction was 4.8 Gy, 36% of the mean baseline MD_Heart_ value. Reductions were larger for patients with greater PTV/heart overlaps (Fig. 2d).

These reductions were achieved without lessening prescribed doses or exceeding protocol dose-volume limits. Tumour coverage measures remained within protocol limits, with small though statistically significant losses (Table 1). For D_PTV-90%_, the percentage of prescribed dose covering 90% of the PTV, the median (range) was 98.4% (95.3%, 99.0%) at baseline versus 97.9% (95.7, 98.9%) after re-optimization (*p* < 0.001). There was a small but significant change in values of EQD2_Lung-mean_, the mean equivalent dose in 2 Gy fractions across the both lungs minus iGTV (*p *= 0.03, Table 2): specifically, the median.

**Table 1:** Median values (ranges) of CTV and PTV coverage measures at baseline and after re-optimization, and two-sided significances of changes in distributions of these measures following re-optimization

Coverage measure (IDEAL-CRT limit)	At baseline	After MD_Heart_ reduction	After V_Heart-50-Gy_ reduction	After V_LAwall-63-Gy_ reduction
D_PTV-90%_* (≥ 95% prescribed dose)	98.6% (96.7, 99.0%)	97.9% (95.7, 98.9%) *p* = 6 × 10^–4^	98.0% (96.3, 98.9%) *p* = 2 × 10^–4^	98.4% (95.3, 99.0%) *p* = 0.31
D_PTV-98%_ (≥ 90% prescribed dose)	96.7% (92.4, 97.9%)	95.0% (90.7, 97.3%) *p* = 4 × 10^–4^	95.1% (91.5, 97.5%) *p* = 2 × 10^–4^	96.4% (90.4, 97.7%) *p* = 0.09
D_CTV-99%_ (≥ 95% prescribed dose)	98.0% (96.5, 98.6%)	97.6% (95.7, 98.4%) *p* = 7 × 10^–3^	97.7% (96.1, 98.4%) *p* = 8 × 10^–3^	97.9% (95.6, 98.6%) *p* = 0.27
D_PTV-99.5%_ (non-protocol measure)	95.2% (86.5, 96.9%)	91.9% (86.7, 96.4%) *p* = 1 × 10^–3^	92.5% (86.0, 96.4%) *p* = 3 × 10^–4^	94.6% (86.9, 96.6%) *p* = 0.09

^*^D_Structure-X%_ denotes the minimum percentage of the prescribed dose covering the most highly irradiated X% of a structure

**Table 2:** Median values (ranges) of measures of irradiation of both lungs excluding iGTV, at baseline and after re-optimization, and two-sided significances of changes in distributions of these measures following re-optimization

Dose-volume measure	At baseline	After MD_Heart_ reduction	After V_Heart-50-Gy_ reduction	After V_LAwall-63-Gy_ reduction
EQD2_Lung-mean_*	13.7 Gy (8.0, 16.8 Gy)	13.9 Gy (7.2, 16.6 Gy) *p* = 0.03	13.7 Gy (7.7, 16.7 Gy) *p* = 0.07	14.1 Gy (8.0, 16.8 Gy) *p* = 0.86
Mean lung dose	15.7 Gy (9.4, 19.5 Gy)	15.8 Gy (8.5, 19.3 Gy) *p* = 0.03	15.7 Gy (9.1, 19.4 Gy) *p* = 0.06	16.0 Gy (9.4, 19.5 Gy) *p* = 0.89
V_Lung-10-Gy_**	47.2% (29.0,70.9%)	46.3% (27.6, 73.9%) *p* = 5 × 10^–3^	46.5% (29.0, 76.5%) *p* = 0.16	47.5% (29.0, 77.4%) *p* = 0.88
V_Lung-20-Gy_	27.2% (15.6, 34.4%)	28.1% (13.6, 34.8%) *p* = 0.98	26.4% (14.9, 34.5%) *p* = 0.30	26.4% (15.6, 34.9%) *p* = 0.94
V_Lung-30-Gy_	17.1% (7.9, 23.7%)	16.4% (6.7, 24.5%) *p* = 0.59	16.7% (7.7, 23.7%) *p* = 8 × 10^–3^	16.6% (7.9, 23.7%) *p* = 0.72
V_Lung-50-Gy_	7.0% (2.6, 12.2%)	6.9% (2.2, 14.4%) *p* = 1.00	7.0% (2.4, 12.6%) *p* = 0.05	7.5% (2.6, 12.2%) *p* = 0.30

^*^Equivalent dose in 2 Gy fractions averaged across both lungs minus iGTV

^**^The fraction of both lungs minus iGTV receiving ≥ 10 Gy

EQD2_Lung-mean_ rose from 13.7 Gy by 0.2 Gy following re-optimization, but the average fell by 0.4 Gy. Numbers of patients with dose-volume metrics lying within 10% of each non-cardiac protocol OAR limit were unchanged after re-optimization for three limits, and rose by one patient each for three limits (Additional file 1: Table S4).

### ***Purposeful V***_***Heart-50-Gy***_*** reductions***

Values of V_Heart-50-Gy_ in baseline plans and plans re-optimized to reduce V_Heart-50-Gy_ are plotted in Fig. 2b. The average V_Heart-50-Gy_ reduction was 2.2% absolute, 40% of the mean baseline value. Tumour coverage losses were small though significant (Table 1). The median EQD2_Lung-mean_ value following re-optimization was unchanged from baseline (Table 2). Numbers of patients with dose-volume metrics lying within 10% of each OAR limit changed little following re-optimization (Additional file 1: Table S4).

### ***Purposeful V***_***LAwall-63-Gy***_*** reductions***

V_LAwall-63-Gy_ values are plotted in Fig. 2c. The average V_LAwall-63-Gy_ reduction was 2.4% absolute, 96% of the mean baseline value. Tumour coverage losses were small and insignificant (Table 1). EQD2_Lung-mean_ values were insignificantly larger after re-optimization, the median value rising by 0.4 Gy (Table 2). Numbers of patients with dose-volume metrics lying within 10% of each OAR limit were unchanged following re-optimization for three limits, and rose by one patient for two limits and by two patients for one limit (Additional file 1: Table S4).

### Knock-on reductions

**Fig. 3:**
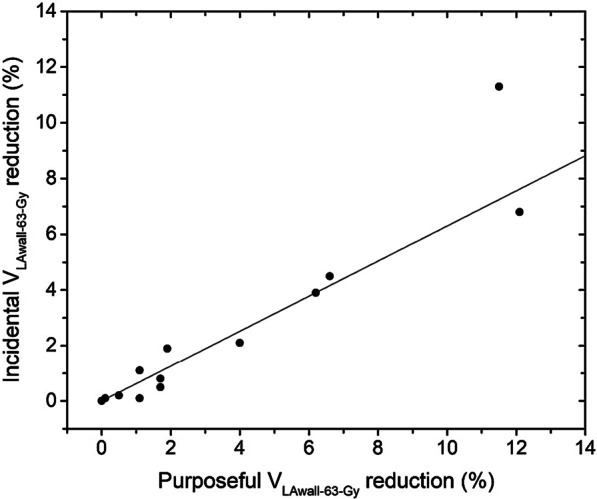
Knock-on versus purposefully-achieved V_LAwall-63-Gy_ reductions. The knock-on V_LAwall-63-Gy_ reductions were achieved in the course of purposefully reducing MD_Heart_ values. The plotted line represents knock-on reductions as 63% of purposeful reductions

Knock-on reductions in MD_Heart_, V_Heart-50-Gy_ and V_LAwall-63-Gy_ made when purposefully reducing others of these measures are summarized in Table 3. Purposeful reduction of MD_Heart_ led to the greatest average knock-on reductions, amounting to 107% and 68% of the purposefully-achieved reductions for V_Heart-50-Gy_ and V_LAwall-63-Gy_. The knock-on V_LAwall-63-Gy_ reductions accompanying purposeful MD_Heart_ reductions are plotted against purposefully-made V_LAwall-63-Gy_ reductions in Fig. 3.

**Table 3:** Average knock-on reductions in cardiac irradiation measures (II) made when other measures (I) were purposefully reduced, compared to average reductions made in the second measure (II) when it was purposefully reduced

Measure I	Measure II	Average knock-on reduction in measure II	Average purposeful reduction in measure II	Ratio of average knock-on and purposeful reductions in measure II
MD_Heart_	V_Heart-50-Gy_	2.34%	2.19%	1.07
MD_Heart_	V_LAwall-63-Gy_	1.65%	2.43%	0.68
V_Heart-50-Gy_	MD_Heart_	1.82 Gy	4.76 Gy	0.38
V_Heart-50-Gy_	V_LAwall-63-Gy_	1.54%	2.43%	0.63
V_LAwall-63-Gy_	MD_Heart_	0.55 Gy	4.76 Gy	0.12
V_LAwall-63-Gy_	V_Heart-50-Gy_	0.77%	2.19%	0.35

Knock-on reductions in a panel of further measures that resulted from purposeful MD_Heart_ reduction have also been determined (Table 4). Average knock-on reductions in mean doses to the LA wall, right atrium, left and right ventricles, right coronary artery, aortic valve region and ascending aorta were 26–52% of baseline values, and the average reduction in the volume of the aortic valve region receiving 35–43 Gy was 100%.

**Table 4:** Average knock-on reductions in other cardiac irradiation measures when MD_Heart_ was purposefully reduced, as a fraction of mean baseline values

Irradiation measure	Structure	Mean value at baseline	Mean value after MD_Heart_ reduction	Mean knock-on reduction	Fractional mean knock-on reduction (%)
Mean dose	LA wall	17.5 Gy	12.1 Gy	5.4 Gy	31
	Right atrium	12.5 Gy	8.8 Gy	4.7 Gy	38
	Left ventricle	7.3 Gy	4.3 Gy	3.0 Gy	41
	Right ventricle	7.0 Gy	3.4 Gy	3.6 Gy	51
	AVR*	13.8 Gy	6.7 Gy	7.1 Gy	51
	Right CA^†^	11.8 Gy	5.9 Gy	5.9 Gy	50
	Ascending aorta	27.7 Gy	20.4 Gy	7.3 Gy	26
Max dose	Right atrium	34.2 Gy	27.0 Gy	7.2 Gy	21
	Left ventricle	29.5 Gy	23.3 Gy	6.2 Gy	21
	Right ventricle	23.6 Gy	15.4 Gy	8.2 Gy	35
	Right CA	15.5 Gy	9.4 Gy	6.1 Gy	39
	Ascending aorta	61.2 Gy	59.3 Gy	1.9 Gy	3
V_LAwall-63-Gy_	LA wall	2.5%	0.8%	1.7%	68
V_AVR-35–43-Gy_**	AVR	7.3%	0%	7.3%	100

^*^Aortic valve region

^†^Coronary artery

^**^Fractional volume of the AVR receiving 35–43 Gy

Average knock-on reductions in maximum doses to the right atrium, ventricles and right coronary artery were 21–47% of baseline values, but for the ascending aorta the maximum dose was reduced by an average of only 3%. In 14 of the patients studied maximum doses to the ascending aorta were similar to prescribed tumour doses and were located in the section of the vessel lying immediately above the heart. Detailed investigation of the plans of two of these patients showed that only small reductions in volumes of the ascending aorta receiving doses in excess of thresholds between 60 and 95% of the prescribed dose could be achieved by lowering the mean heart dose. However, when the ascending aorta was merged with the heart and the mean dose to this composite structure was reduced these high-dose ascending aorta volumes fell much more, by 35–56% of their baseline values, although maximum doses still fell little.

### Trial patient numbers

The mean purposefully-achieved reductions in MD_Heart_, V_Heart-50-Gy_ and V_LAwall-63-Gy_ translate into expected HRs for all-cause death of 0.806, 0.943 and 0.838. Based on these HRs, 1:1 randomized trials designed to test improved OS with a 5% type-I error-rate and 80% power would need 1140, 14,850 or 1798 patients.

Particularly large mean reductions in cardiac dose-volume measures were achieved for patients with baseline values exceeding median values in published series. For the 8 patients with baseline MD_Heart_ > 11 Gy the average reduction in this measure was 8.8 Gy. Similarly, for the 8 patients with V_Heart-50-Gy_ > 4%, the mean V_Heart-50-Gy_ reduction was 4.4%; and for the 5 patients with baseline V_LAwall-63-Gy_ > 2.2%, the mean V_LAwall-63-Gy_ reduction was 8.1%. These reductions correspond to HRs of 0.672, 0.887 and 0.551, based on which 359, 3604 or 170 patients would be needed in trials recruiting from these subpopulations alone.

## Discussion

In baseline plans median values of D_Heart-100%,_ D_Heart-67%_ and D_Heart-33%_, the minimum doses covering 100%, 67% and 33% of the heart, were just 1.3%, 3.6% and 7.5% of their IDEAL-CRT limits. Other groups have reported similar findings [2, 7], showing that typically-used limits do not effectively restrict heart doses during treatment planning. By adding extra optimization objectives mean values of MD_Heart_, V_Heart-50-Gy_ and V_LAwall-63-Gy_ were reduced by 36%, 40% and 96% relative to baseline values, without breaching protocol limits on irradiation of other normal tissues or minimum tumour coverage requirements.

For patients treated for breast cancer, the risk of major coronary events following RT has previously been shown to rise linearly with MD_Heart_ [9]. However, the scale of associations between survival and MD_Heart_ seen in patients treated for LA-NSCLC is greater than expected from the breast RT data. For example, prescribed tumour doses were 16% greater in the high-dose arm of RTOG-0617 than in the low-dose arm, an increase that would have raised MD_Heart_ by an average of roughly 2 Gy. The breast RT data indicates that for a 50 year-old woman with one or more cardiac risk factors, this 2 Gy rise in MD_Heart_ would increase the risk of death from ischaemic heart disease by only around 0.5% absolute [9], and yet in the high-dose arm of RTOG-0617 2-year OS was 13% absolute less than in the low-dose arm. This seemingly greater effect of MD_Heart_ on survival in lung cancer patients needs to be weighed against the detrimental effects of cardiac-sparing seen in our study, namely small increases in numbers of patients with dose-volume metrics lying within 10% of dose-limits, and small reductions in PTV coverage. Net survival gains from cardiac sparing could be tested most clearly in randomized trials, but large numbers of patients would be needed: we estimate 1,140 or 1,798 assuming that OS is causally linked to MD_Heart_ or V_LAwall-63-Gy_, or 14,850 if OS is causally related to V_Heart-50-Gy_.

The survival benefit predicted for cardiac-sparing RT is derived largely from patients with baseline heart doses greater than median values in patient series (Fig. 2). For these patients cardiac irradiation can be reduced more, leading to larger predicted survival increases of 13%, 18% or 4% from a 50% level if OS is causally related to MD_Heart_, V_LAwall-63-Gy_ or V_Heart-50-Gy_. These increases could be tested more efficiently in trials enriched for such patients [20, 21], who could be identified at baseline planning. Estimated numbers of patients required are notably smaller than for the wider population: 359 or 170 if OS is causally related to MD_Heart_ or V_LAwall-63-Gy_, or 3,604 if related to V_Heart-50-Gy_. Because the dose-volume thresholds used for patient selection are published median values, roughly double these numbers would need to be screened, around 700 patients for a study based on MD_Heart_ reduction, making the logistics challenging. Enrichment strategies have been used in trials evaluating treatments in subpopulations positive for biomarkers. Heart irradiation could act as one such biomarker, potentially allowing trialling to be embedded within a larger umbrella study testing treatments for several biomarker-defined subpopulations [22].

As the cardiac dose-volume measure most strongly associated with survival remains to be identified, we have checked the robustness of cardiac-sparing to the possibility that the measure being reduced is not the key one determining survival. Of the three measures purposefully reduced, V_LAwall-63-Gy_ could be decreased most completely. However, its purposeful reduction led to relatively small knock-on reductions in the whole-heart measures. Purposeful reduction of MD_Heart_ was the most robust option explored: overall it offered the greatest predicted survival benefits, and provided large knock-on reductions in V_Heart-50-Gy_ and V_LAwall-63-Gy_, and useful knock-on reductions in a panel of other cardiac irradiation measures reported to be associated with OS. Because the upper section of the ascending aorta lies above the top of the atlas-defined heart volume, maximum doses in this structure were reduced less via mean heart dose reduction. If considered important, however, high-dose volumes of the ascending aorta can be reduced to a greater extent by adding this structure to the heart and reducing the mean dose to the composite volume.

The commonly used target coverage measures reported here fell little as heart doses were reduced (Table 1), although doses within the small PTV/LA wall overlap region sometimes fell more appreciably (Fig. 1). To check this further we collected values for D_PTV-99.5%_, the percentage of the prescribed dose covering 99.5% of the PTV. The greatest median decrease in D_PTV-99.5%_ was 3.3%, following reduction of MD_Heart_ (Table 1). Such a dose reduction right across the PTV might lessen 2-year OS by 2–4% [23], but the same reduction in D_PTV-99.5%_ should diminish survival much less, because the PTV sub-volumes involved are small (0.5%) [24] and located at the PTV edge where tumour cell density is lower [25].

Cardiac-sparing had little impact on lung irradiation-levels (Table 2), a finding that can be explained straightforwardly. The heart lies quite centrally within the lungs, which are much larger, with a typical total volume of 6 versus 0.35 L [26, 27]. Consequently, even if all the radiation fluence removed from the heart was redistributed to the lungs, the lung mean dose would rise considerably less than the mean heart dose would fall.

Ferris et al. recently reported that heart doses in cardiac-optimized VMAT plans created retrospectively for LA-NSCLC patients treated in 2013–2017 were lower than in the original plans used to treat patients, but could not establish how much this improvement owed to enhanced planning software, increasingly skilled planners, cardiac substructure outlining, or intentional heart-sparing [28]. In our study, the same treatment planner (LT) contemporaneously created baseline and cardiac-sparing plans using the same software, and therefore the reduced heart doses were a direct consequence of objectives added to the optimization process to reduce cardiac irradiation-levels.

Our study is limited to 20 patients with a 50:50 split of left- and right-sided tumours and a 60:40 IIIA/IIIB stage-split, similar to the 65:35 split in RTOG-0617. Subject to achieving these splits, patients were drawn from a contiguous series treated at CCC, expected to represent the wider patient population. The 13.2 Gy average value of mean heart dose in the baseline plans created for these patients is comparable to means of 11.6 and 17.0 Gy reported for series of 78 and 35 LA-NSCLC patients respectively [12, 29], and medians of 11.0 and 16.6 Gy reported for 125 and 468 patients [10, 30]. In the ongoing RTOG-1308 trial of proton versus photon radiotherapy for LA-NSCLC, D_Heart-35%_ and D_Heart-50%_ were limited to 45 Gy and 30 Gy, tighter constraints than typically set [31]. The highest D_Heart-33%_ and D_Heart-50%_ values in our baseline plans were 43.1 Gy and 30.1 Gy, and therefore the tighter RTOG-1308 limits would have negligibly lessened the heart doses in the baseline plans, or the gains achieved via re-optimization.

## Conclusions

Heart doses in photon beam RT treatments of LA-NSCLC could be substantially reduced without markedly compromising tumour dose coverage or raising dose-levels in other OARs. In a cohort of 20 routinely-treated patients retrospectively re-planned according to the isotoxic IDEAL-CRT protocol, the average reductions achieved in MD_Heart_, V_Heart-50-Gy_ and V_LAwall-63-Gy_ were 4.8 Gy, 2.2% and 2.4% absolute. Purposeful reduction of MD_Heart_ provided useful knock-on reductions in V_Heart-50-Gy_, V_LAwall-63-Gy_ and a basket of other measures of cardiac irradiation, insuring against the possibility that these measures are more directly related to survival changes than is MD_Heart_.

The average purposeful reductions in MD_Heart_, V_Heart-50-Gy_ and V_LAwall-63-Gy_ translated to predicted OS gains that would require many patients to test in a randomized trial. Average reductions in mean heart doses were larger in subgroups of patients with baseline levels of cardiac irradiation greater than median values in published series, potentially permitting trialling in 359 patients enriched for greater baseline MD_Heart_ values.

## Supplementary Information

**Additional file 1.** Supplementary Materials.

## Data Availability

Available on request from LT.

## Abbreviations

AIP: Average intensity projection
CCC: Clatterbridge Cancer Centre
CRT: Chemoradiotherapy
CT: Computed tomography
D_Heart-XX%_: Minimum dose to the most highly irradiated XX% of heart
D_PTV-XX%_: Minimum dose to the most highly irradiated XX% of the PTV
EQD2: Equivalent dose in 2 Gy fractions
EQD2_Lung-mean_: Mean EQD2 in both lungs minus iGTV
iGTV: Internal gross tumour volume
HR: Hazard ratio
LA: Left atrium
LA-NSCLC: Locally advanced non-small cell lung cancer
MD_Heart_: Heart mean dose
OAR: Organ at risk
OS: Overall survival
PTV: Planning target volume
RIHD: Radiation induced heart disease
RT: Radiotherapy
RTOG: Radiation therapy oncology group
V_AVR-35__–__43-Gy_: Fractional volume of aortic valve regions receiving 35–43 Gy
V_Heart-50-Gy_: Fractional heart volume receiving ≥ 50 Gy
V_LAwall-63-Gy_: Fractional LA wall volume receiving ≥ 63 Gy
V_Lung-XX-Gy_: Fractional volume of both lungs minus iGTV receiving ≥ XX Gy
VMAT: Volumetric modulated arc therapy
