# Supplementary material for: Cardiac-sparing radiotherapy for locally advanced non-small cell lung cancer
Source: Radiat Oncol. 2021 Jun 3;16:95. doi: 10.1186/s13014-021-01824-3 (PMC8176693; doi:10.1186/s13014-021-01824-3)
Supplement: Supplementary file 1 — Additional file 1. Supplementary Materials. [file 13014_2021_1824_MOESM1_ESM.docx]

**Supplementary Information**

Supplementary Table 1. Stage, prescribed dose and geometric characteristics for the 20 patients studied.

| Tumour laterality | Stage | Prescribed dose  (Gy) | iGTV volume  (cm^3^) | PTV/Heartoverlap (cm^3^) | CTV/Heart overlap (cm^3^) | PTV/LA wall overlap (cm^3^) |
| --- | --- | --- | --- | --- | --- | --- |
| R | 3A | 71.6 | 7.4 | 1.5 | 0.1 | 0 |
| R | 3A | 71.6 | 20.7 | 9.4 | 4.6 | 0 |
| R | 3A | 68.8 | 21.2 | 4.3 | 0.7 | 0 |
| R | 3A | 63.0 | 102.3 | 31.3 | 12.0 | 4.1 |
| R | 3A | 63.5 | 179.6 | 20.9 | 7.5 | 2.4 |
| R | 3A | 66.0 | 199.4 | 7.1 | 0.2 | 0.2 |
| R | 3B | 68.0 | 121.0 | 2.7 | 0 | 0.6 |
| R | 3B | 68.0 | 122.0 | 10.6 | 2.2 | 0.1 |
| R | 3B | 68.8 | 128.6 | 39.4 | 19.9 | 3.9 |
| R | 3B | 63.0 | 243.2 | 3.9 | 0.8 | 0 |
| L | 3A | 73.0 | 17.3 | 14.5 | 5.4 | 0 |
| L | 3A | 73.0 | 89.1 | 13.8 | 1.4 | 0.1 |
| L | 3A | 63.0 | 111.0 | 42.3 | 15.9 | 1.4 |
| L | 3A | 68.8 | 124.8 | 5.1 | 0.9 | 1.6 |
| L | 3A | 67.4 | 126.9 | 1.0 | 0.1 | 0 |
| L | 3A | 63.0 | 191.9 | 15.8 | 4.6 | 3.3 |
| L | 3B | 68.8 | 41.3 | 6.3 | 1.1 | 0 |
| L | 3B | 68.8 | 51.3 | 0 | 0 | 0 |
| L | 3B | 68.8 | 100.9 | 7.1 | 1.0 | 0 |
| L | 3B | 68.8 | 176.5 | 10.8 | 2.7 | 0 |

Supplementary Table 2. IDEAL-CRT dose-volume limits for OARs, PTV and CTV.

| Structure | Limit |
| --- | --- |
| Heart | D_Heart-100%_  ≤ 45 Gy, D_Heart-67%_ ≤ 53 Gy, D_Heart-33%_ ≤ 60 Gy |
| Both lungs minus iGTV | EQD2_Lung-mean_ ≤ 16.5 Gy*, V_Lung-20-Gy_ ≤ 35% |
| Spinal cord^✝^ | D_Cord-0.1cc_ ≤ 47 Gy |
| Brachial plexus | D_BP-30%_ ≤ 60 Gy, D_BP-0.1cc_ ≤ 65 Gy |
| Oesophagus | D_Oesoph-1.0cc_ ≤ 68 Gy |
| Proximal bronchial tree | D_PBT-1.0cc_ ≤ 73 Gy |
| PTV | D_PTV-98%_ ≥ 90% prescribed dose |
| PTV | D_PTV-90%_ ≥ 95% prescribed dose |
| CTV | D_CTV-99%_ ≥ 95% prescribed dose |

D_Structure-X[cc or %]_ denotes the minimum dose delivered to the most highly irradiated X cc or X% of a structure. V_Structure-X-Gy_ denotes the percentage volume of a structure receiving more than X Gy.

* If a median tumour dose of 63 Gy could not be prescribed for an IDEAL-CRT patient without exceeding 16.5 Gy EQD2_Lung-mean_, then 63 Gy was still prescribed provided EQD2_Lung-mean_ ≤ 19.2 Gy and V_Lung-20-Gy_ ≤ 35%.

^✝^ A 3 mm margin was added to form a planning organ-at-risk volume.

Supplementary Table 3. Values of conventional heart dose-volume measures in patients’ baseline plans, listed in the order of Table 1, together with medians, ranges and limits set in IDEAL-CRT.

| D_Heart-100%_ (Gy)  (limit = 45 Gy) | D_Heart-67%_ (Gy)  (limit = 53 Gy) | D_Heart-33%_ (Gy)  (limit = 60 Gy) |
| --- | --- | --- |
| 0.1 | 0.4 | 1.1 |
| 0.2 | 0.7 | 2.1 |
| 0.4 | 1.0 | 2.6 |
| 10.3 | 23.2 | 36.7 |
| 0.5 | 7.9 | 19.6 |
| 7.1 | 23.6 | 43.1 |
| 0.7 | 8.7 | 20.7 |
| 0.6 | 2.0 | 6.4 |
| 1.0 | 15.6 | 28.5 |
| 0.6 | 2.0 | 5.6 |
| 0.4 | 1.2 | 2.9 |
| 0.5 | 1.7 | 4.5 |
| 8.2 | 22.3 | 35.0 |
| 0.2 | 0.7 | 2.1 |
| 0.7 | 2.0 | 4.4 |
| 1.5 | 15.4 | 27.3 |
| 0.5 | 1.3 | 2.9 |
| 0.3 | 0.6 | 0.9 |
| 0.4 | 1.1 | 2.5 |
| 0.6 | 1.6 | 3.8 |
|  | Median (range) (Gy) |  |
| 0.6 (0.1, 10.3) | 1.9 (0.4, 23.6) | 4.5 (0.9,43.1) |

Supplementary Table 4. Numbers of patients for whom individual OAR dose-volume measures approached
within 10% of their IDEAL-CRT limits. All limits were met in all plans.

| OAR dose-volume limit | At baseline | After MD_Heart_ reduction | After V_Heart-50-Gy_ reduction | After V_LAwall-63-Gy_ reduction |
| --- | --- | --- | --- | --- |
| D_Cord-0.1cc_ ≤ 47 Gy | N = 6 | 7 | 5 | 6 |
| D_BP-0.1cc_ ≤ 65 Gy | N = 0 | 0 | 0 | 0 |
| D_Oesoph-1.0cc_ ≤ 68 Gy | N = 12 | 13 | 13 | 13 |
| D_PBT-1.0cc_ ≤ 73 Gy | N = 13 | 13 | 13 | 15 |
| V_Lung-20-Gy_ ≤ 35% | N = 4 | 5 | 5 | 5 |
| EQD2_Lung-mean_ ≤ 19.2 Gy | N = 0 | 0 | 0 | 0 |
